# Comparison of oral versus intravenous glucose exposure on plasma growth hormone levels: a crossover study in healthy volunteers

**DOI:** 10.1007/s11102-025-01633-x

**Published:** 2026-01-12

**Authors:** Anna Katarina Vinten, Nanna Thurmann Jørgensen, Esben Budtz-Jørgensen, Marianne Klose, Mikkel Andreassen

**Affiliations:** 1 Department of Nephrology and Endocrinology, Rigshospitalet, Copenhagen, Denmark; 2 Department of Clinical Medicine, Faculty of Health and Medical Sciences, University of Copenhagen, Copenhagen, Denmark; 3 Section of Biostatistics, Department of Public Health, Faculty of Health Sciences, University of Copenhagen, Copenhagen, Denmark

**Keywords:** Growth hormone, Oral glucose tolerance test, Intravenous glucose infusion, GLP-1 (glucagon-like peptide-1), GIP (glucose-dependent insulinotropic polypeptide), Incretin, Insulin

## Abstract

**Background:**

Hypoglycaemia stimulates growth hormone (GH) secretion, whereas hyperglycaemia suppresses it. However, the underlying mechanisms are not fully understood, particularly the potential role of gut-derived hormones released in response to oral glucose.

**Aim:**

To investigate whether GH suppression is modulated by the route of glucose administration.

**Methods:**

A two-day intervention study in healthy volunteers. GH, insulin, glucagon-like peptide-1 (GLP-1), and glucose-dependent insulinotropic polypeptide (GIP) responses during a 2-h oral glucose tolerance test (OGTT) were compared with those during a 2-h isoglycaemic intravenous (IV) glucose infusion. GH levels were analyzed using paired t-test of GH concentrations at every blood sample time point. The effect of intervention on all measured hormones were also assessed by paired t-test of Area Under the Curve (AUC).

**Results:**

12 healthy volunteers (6 females, mean age 47.9 ± 5.4 years) were included. In 9 of the 12 subjects, IV glucose induced an early peak in plasma-GH followed by a decrease. At 20 min after glucose intake GH levels increased by 46% during IV glucose compared to a decrease of 17% during oral glucose. The biggest numerically difference in GH between oral vs IV glucose was seen at 45 min (median [range], 0.30 [0.05–1.13] vs. 0.46 [0.05–9.82] µg/l, p = 0.072). There was no difference between AUC of GH levels (p = 0.381). During IV glucose, two subjects did not reach the threshold for excluding acromegaly. Oral glucose showed significant increases compared to IV glucose for insulin (p < 0.001), GLP-1 (p = 0.002) and GIP (p < 0.001) when using paired t-test of AUC.

**Conclusions:**

Route of glucose exposure might influence the suppressive effect of glucose on GH secretion. This finding suggests that stimulation of other hormone systems may play a contributing role on the regulation of GH. The potential mechanism behind remains elusive but changes in gut-derived hormones might be of importance.

## Introduction

Growth Hormone(GH) is an anabolic hormone that plays a key role in the regulation of blood glucose. Hypoglycaemia stimulates GH secretion whereas hyperglycaemia inhibits it [1]. Consequently, glucose is used in the diagnosis of acromegaly to document autonomous GH secretion. The diagnosis is based on inadequate suppression of GH during an Oral Glucose Tolerance Test (OGTT) [1].

The exact mechanism responsible for the suppression of GH secretion remains unclear. A prevailing hypothesis suggests that glucose stimulates somatostatin release from the hypothalamus, which in turn inhibits somatotroph activity in the pituitary gland [1]. In addition, a concomitant decrease in ghrelin secretion might also contribute to reduced GH levels during glucose exposure.

Within the last 30 years it has become evident that hormones derived from the gastrointestinal tract (GI-hormones) play a major role in metabolic homeostasis, body weight and hunger. In particular, the incretin hormones, glucose dependent insulinotropic peptide (GIP) and glucagon like peptide-1 (GLP-1), secreted from the small intestine have been subject of extensive research. Except for ghrelin, GI-hormones have not been shown to be involved in regulation of pituitary hormones during normal physiology. However, it has recently been found that GLP-1 receptor agonists (GLP-1 RA) have a stimulatory effect of GH secretion [2]. In addition, a subset of acromegalic patients – approximately 30% – show a paradoxical increase in GH levels during glucose exposure [3]. This phenomenon has been attributed to an increased number of GIP receptors on GH secreting tumor cells thereby linking GH secretion with GI-hormones [3–5].

The question of whether the route of glucose administration affects the GH suppressive effect of glucose has not been sufficiently investigated. The aim of this study was to compare GH levels during a OGTT with an isoglycaemic intravenous (IV) infusion of glucose in healthy subjects.

## Subjects and methods

The subjects consisted of healthy volunteers (n = 12), who had previously served as the control group in a published study [6]. The subjects were recruited through an online platform. The exclusion criteria were conditions related to glucose metabolism and gastrointestinal function, including diabetes, use of diabetogenic medications, acute or chronic pancreatitis, inflammatory bowel disease, major gastrointestinal surgery, pregnancy, breastfeeding and active cancer. Prior to testing, all subjects were screened, with assessment of height, weight, and plasma hemoglobin A1 [6]. Two weeks before testing, 6 of the 12 subjects started temporarily daily hydrocortisone treatment until the end of the last test day to serve as controls for patients with acromegaly on hydrocortisone in the original study [6].

The subjects completed two glucose tolerance tests on separate days: a 3-h OGTT with 75 g oral glucose on day 1, and an isoglycaemic intravenous infusion of 20% glucose at adjustable rates on day 2 [6]. On day 2 a manual glucose infusion pump was used by adjusting the infusion rate to mimic response of the individual subjects during the OGTT using blood sample measurements from day 1. Both tests were performed following an overnight fast of 10 h and began between 8.00 and 10.00 in the morning [6]. An intravenous cannula was placed into the forearm vein, and the hand was heated by a 50 degree-heating pad to ‘arterialize’ collected venous blood. Blood samples were taken at fixed time points from − 15 to 180 min [6]. To enable comparison with a standard OGTT for diagnosing acromegaly, the time interval in the present study was limited to 0 to 120 min.

Blood samples were analyzed for plasma-glucose, -GH, -insulin, -GLP-1 and -GIP. Data for plasma-glucose, -insulin, -GLP-1 and -GIP during oral glucose were also presented in the original paper (control subjects) [6]. All GH measurement as well as levels of glucose and other hormones during IV glucose have not previously been published. Levels of glucose (mmol/l) were measured every 5 min by collection of samples in pico tubes. Fasting concentrations of plasma glucose were calculated from the mean of the three blood samples drawn at − 15, − 10 and 0 min. Samples used for analyzing levels of GH were collected in chilled tubes containing Li-heparin. Obtained blood samples were cooled on ice, centrifuged (1200 × g for 20 min at 4 °C) and stored at − 20 °C after collection until analysis [6].

## Biochemical analyses

Plasma glucose concentration was measured using the glucose oxidase method by an YSI (YSI 2300 STAT plus analyzer; Yellow Springs Instruments, Yellow Springs, OH, USA). The measurements were run immediately after sampling during OGTT and isoglycaemic IV glucose infusion. The within-run precision was calibrated using an YSI glucose standard between each glucose measurement [6]. Samples for levels of hormones were measured with as few assay-runs as possible [6].

Plasma concentrations of GH were measured using the sandwich electrochemiluminescence immunoassay (ECLIA) performed on a Cobas pro e801 (Roche Diagnostics GmbH, Switzerland). The local, long-term maximal total assay coefficient of variation (CV_max_) of GH was 6% at concentrations of both 2.13 and 28.8 µg/l. The detection limit of the GH-assay was 0.05 µg/l.

Plasma concentrations of insulin were measured as previously described [6]. The local, long-term maximal total assay coefficient of variation (CV_max_) of insulin was 5% at concentrations of both 200 and 1000 pmol/l. The detection limit of the insulin assay was 1.39 pmol/l [6]. Plasma concentrations of GIP and GLP-1 were measured as previously described [6] including plasma extraction [7, 8]. Both assays had sensitivity below 1 pmol/l and an intraassay coefficient of variation below 6% at 20 pmol/l. Recovery of calibrator added to plasma before extraction was about 100% when corrected for losses inherent in the plasma extraction procedure [6].

## Ethics

All subjects gave their written informed consent and voluntarily participated in the study. The protocol was approved by Ethics Committee (H-15021390), Capital Region of Denmark (Hillerod, Denmark) and the Danish Data Protection Agency and registered at ClinicalTrials.gov (ID: NCT02005978). The study followed the Declaration of Helsinki [6].

## Statistical analyses

Statistical analyses were made in IBM SPSS Statistics version 29.0.2.0. Results are expressed as mean ± SD when normally distributed and as median [range] when not normally distributed. Normality assumptions were verified visually. Missing values were imputed by linear interpolation between adjacent measured values.

Values below the lower limit of detection (LOD) were set to the LOD. Non-normally distributed variables were log-transformed (natural logarithm) to meet the assumptions required for parametric statistical analyses.

For each time point, paired t-test were conducted to compare the effect of oral vs. IV glucose on GH levels within subjects. Taking the exponential of the difference on the logarithmic scale yields the ratio of GH levels (geometric means) between the two groups.

The effect of oral vs. IV glucose on concentration of GH and glucose as well as on concentrations of insulin, GLP-1 and GIP were also assessed by paired t-test of Area Under the Curve (AUC) using the trapezoid rule. Time courses of glucose, insulin, GLP-1 and GIP under each condition were plotted over time as mean concentrations.

To illustrate the time course of GH concentrations under each condition, geometric means were calculated. This was done by first applying the natural logarithm to each individual GH value at each time point, computing the mean of the log-transformed values, and then back-transforming (exponentiating) the result to obtain the geometric means. These geometric means were plotted over time to provide a clearer representation of the overall pattern of GH changes in each intervention group. To correct for baseline variability between individuals, GH concentrations were also expressed as relative changes, calculated as the ratio of the value at each time point to the individual’s baseline concentration (ratio = value_t_/baseline).

## Results

F12 subjects (6 females) were included in the study. Mean age was 47.9 ± 5.4 years and BMI was 28.2 ± 3.7 kg/m^2^ (Table 1).

**Table 1:** Antropometric and gluco-metabolic characteristics

	Subjects (n = 12)
Sex (M/F)	6/6
Age (years)	47.9 ± 5.4
Weight (kg)	94.3 [56.1–107.7]
Body Mass Index (kg/m2)	28.2 ± 3.7
Fasting glucose (mmol/l)	5.4 ± 0.7
2 h OGTT glucose (mmol/l)	5.5 [3.2–7.9]

*Results are expressed as mean* ± *S.D. or median [range]. OGTT* = *oral glucose tolerance test*

### Changes in glucose

**Fig. 1:**
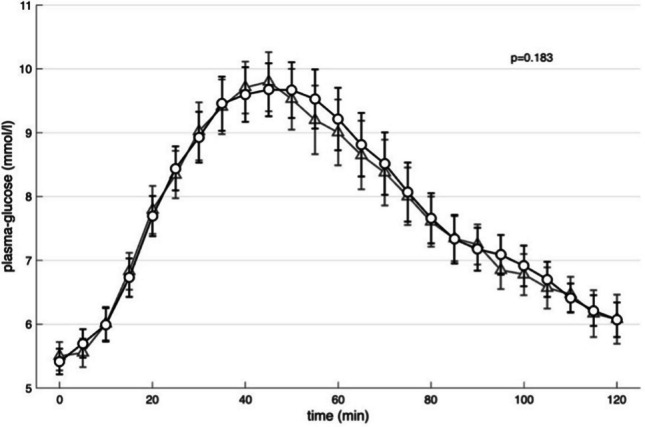
Mean of glucose concentrations response during oral glucose tolerance test (triangle) and isoglycaemic intravenous glucose infusion (circle). p-values and analyses of area under the curve (AUC) was performed using paired t-test. Values are means ± S.E.M

OGTT gave rise to increasing glucose levels with a peak at 45 min (Fig. 1). No significant differences between the administration forms were observed (Fig. 1).

### Changes in GH

**Fig. 2:**
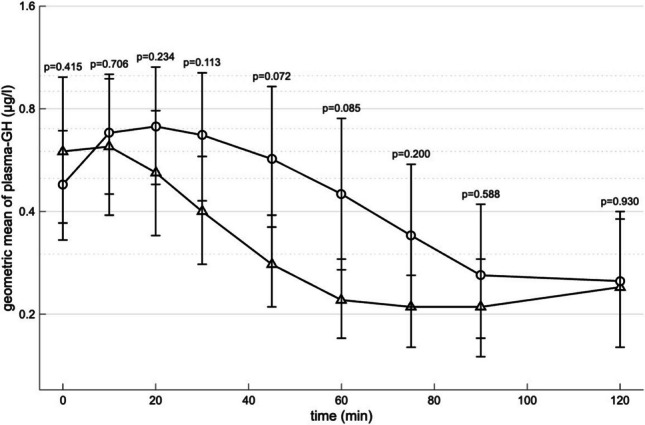
Geometric mean of growth hormone (GH) concentrations response on logarithmic scale during oral glucose tolerance test (triangle) and isoglycaemic intravenous glucose infusion (circle) with p-values of paired testing of GH concentrations at every blood sample time point. p-values was performed using paired t-test. Values are means ± S.E.M

Time-course of GH levels during oral vs. IV glucose are shown in Fig. 2 and in Table 2. Oral glucose load was followed by a decrease in GH levels (Table 2 and Fig. 2). By contrast, parenteral glucose load induced an early peak in plasma-GH followed by a decrease in GH levels (Table 2 and Fig. 2). Assessed by paired t-test on logarithmically transformed GH levels at every blood sample time point, the most significant difference in GH between oral vs IV glucose was seen at 45 min (p = 0.072). Here the difference on the log-scale showed that the geometric mean of GH concentrations under IV was increased by a factor of 2.02 (95% CI: 0.93–4.42) compared to IV.

**Table 2:** GH concentrations according to intervention

	GH (µg/l)	
Time (minutes)	OGTT	IIGI
0	0.55 [0.05–7.05]	0.70 [0.05–3.67]
10	0.56 [0.05–5.10]	0.93 [0.05–5.22]
20	0.52 [0.05–3.67]	0.67 [0.06–6.10]
30	0.41 [0.05–2.38]	0.62 [0.05–7.49]
45	0.30 [0.05–1.13]	0.46 [0.05–9.82]
60	0.23 [0.05–0.75]	0.29 [0.05–8.89]
75	0.25 [0.05–0.67]	0.20 [0.05–5.39]
90	0.19 [0.05–2.22]	0.16 [0.05–4.63]
120	0.13 [0.05–5.97]	0.13 [0.05–2.68]

Results are expressed as median [range]

OGTT = oral glucose tolerance test

IIGI = isoglycaemic intravenous glucose infusion

**Fig. 3:**
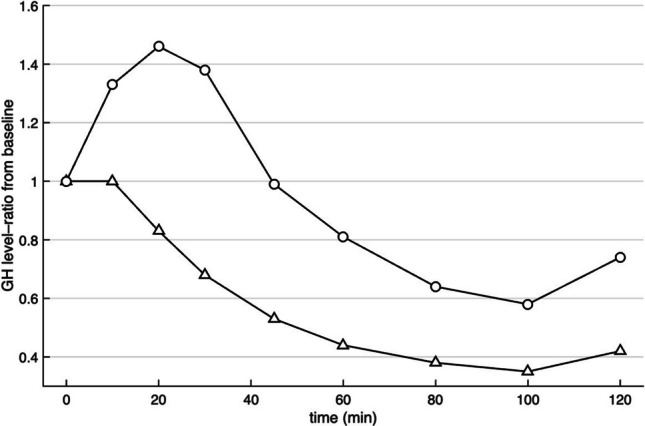
Median of relative changes of growth hormone (GH) concentrations response expressed as ratio = value_t_/baseline during oral glucose tolerance test (triangle) and isoglycaemic intravenous glucose infusion (circle)

Relative changes in GH levels during oral vs. IV glucose are shown in Fig. 3. At 20 min GH levels increased by 46% during IV glucose compared to a decrease of 17% during oral glucose exposure.

**Fig. 4:**
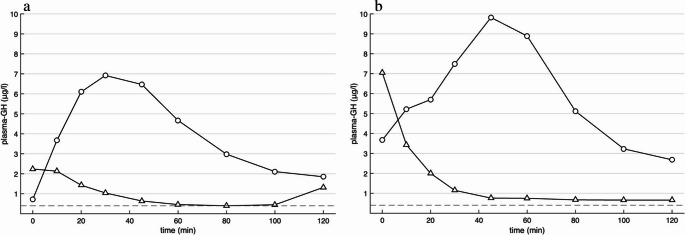
Growth hormone (GH) concentrations for subject that did not reach the threshold (butted, < 0.4 µg/l) for excluding acromegaly during isoglycaemic intravenous glucose infusion (circle, nadir plasma-GH: 0.72 µg/l) (**a**). GH concentrations for subject that did not reach the threshold (butted, < 0.4 µg/l) for excluding acromegaly during oral glucose tolerance test (triangle, nadir plasma-GH: 0.66 µg/l) and isoglycaemic intravenous glucose infusion (circle, nadir plasma-GH: 2.68 µg/l) (**b**)

During isoglycaemic IV glucose infusion, two subjects did not reach the threshold (GH < 0.4 µg/l) for excluding acromegaly (nadir 2.68 and 0.72 µg/l) (Fig. 4) and during OGTT, one participant did not reach the threshold (nadir 0.66 µg/l)(Fig. 4b).

Although, the GH levels was higher for IV at most time points, the difference observed in AUC of GH levels was not statistically significant (p = 0.382).

### Changes in insulin, GLP-1 and GIP

**Fig. 5:**
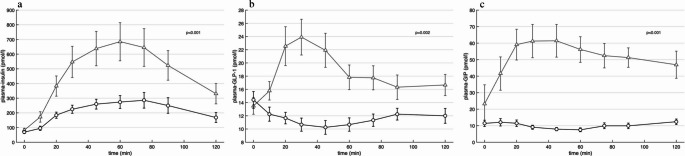
Mean of insulin (**a**), glucagon-like peptide-1 (GLP-1) (**b**) and glucose-dependent insulinotropic polypeptide (GIP) (**c**) concentrations response during oral glucose tolerance test (triangle) and isoglycaemic intravenous glucose infusion (circle). p-values and analyses of area under the curve (AUC) was performed using paired t-test. Values are means ± S.E.M

Changes in levels of insulin, GLP-1 and GIP are shown in Fig. 5. Oral glucose led to significantly higher levels of insulin, GLP-1 and GIP in AUC compared to IV glucose (Fig. 5).

### Missing values of hormones

During OGTT 2/108 values were missing for GH, insulin, and GLP-1, 3/108 values were missing for GIP, and 1/300 values was missing for glucose. During isoglycaemic IV glucose infusion 2/300 values were missing for glucose.

## Discussion

This study gives some support to the hypothesis that route of glucose administration influences the GH-suppressive effect of glucose. Intravenous glucose administration induced a paradoxical early rise in GH levels, followed by persistently higher GH concentrations compared to oral administration. This paradoxical response was observed in 9 of the 12 subjects, and in two subjects the criteria for excluding acromegaly (GH < 0.4 µg/l defined for the OGTT) was not met during IV glucose exposure.

To our knowledge, the effect of oral vs. IV glucose on GH suppression under isoglycaemic conditions has not previously been studied. Aydin et al. examined 18 healthy subjects during OGTT and subsequent IV glucose infusion, reporting higher GH nadirs and generally higher GH levels during IV administration, consistent with our findings [9]. Nakagawa et al. studied eight healthy men and found GH suppression after both oral and IV glucose, but only baseline and 150-min values were reported, and no statistical comparison was made [10]. A major limitation of both studies is that the oral glucose condition was compared with a single IV glucose bolus, which induced a very rapid and pronounced increase in plasma glucose followed by a gradual decline – thus not at all mimicking the plasma glucose profile seen after oral glucose ingestion.

The current hypothesis is that glucose per se stimulates hypothalamic secretion of somatostatin which is a potent inhibitor of GH secretion [11]. In the pituitary, somatostatin receptor-1, -2 and -5 are found to be regulating GH secretion by binding somatostatin [12]. Somatostatin inhibits the GH secretion directly in the pituitary and indirectly by inhibiting growth hormone releasing hormone (GHRH) from the hypothalamus [12]. Besides glucose and GH, somatostatin is stimulated by IGF-1, exercise and immobilization [12]. Based on this glucose-somatostatin mechanism, route of glucose administration should not influence the suppressive effect on GH secretion, and therefore our results suggest that other mechanisms might be involved.

A potential hypothesis is that changes in other GI- and pancreatic hormones might contribute to the changes in GH during oral vs. IV glucose. As reported in multiple prior studies [13, 14], we also found highly significant differences in concentrations of GIP, GLP-1 and insulin dependent on route of glucose administration. The most pronounced difference in GH levels between IV and oral glucose administration occurred within the first 20 min, and within this brief period, significant differences in the levels of insulin, GLP-1 and GIP (oral vs. IV) were already evident (Fig. 5). However, based on the present study it is not possible to draw any conclusions in relation to causality.

Insulin receptors are protein kinase receptors and are found in pituitary tissue in humans [15]. An intervention study from 1997 in 6 healthy volunteers suggested a direct inhibitory effect of insulin on GH-secretion independent of levels of free fatty acids [16]. In support of this hypothesis, in vitro data from mice and baboons showed that insulin directly serve as a suppressor of GH secretion [17, 18]. These in vivo and in vitro results show – in line with our results – reduced inhibition of GH during IV glucose exposure concomitant with reduced increase in insulin compared to oral glucose exposure. In a recent study, GLP-1 receptors were identified in hypothalamic tissue in humans by immunohistochemistry [19], and a PET imaging study identified high uptake of a GLP-1 RA on human pituitary tissue suggesting that GLP-1 receptors are also expressed in the pituitary in humans [20]. The physiological significance of this has not been clarified, but a recently published intervention study in humans showed that the short- and long-acting GLP-1 RAs, exenatide and liraglutide, stimulated GH-secretion [2]. These results are in opposition to our study, which shows reduced levels of GH during high levels of GLP-1. GIP receptors are also expressed in the normal pituitary gland [21], but to our knowledge the role of GIP receptor activation in GH secretion during normal physiology has not been investigated. It should be noted that results obtained in a recent study about acromegaly, shows a stimulatory effect of GIP [3], which is also in opposition to what was found in the present study, where a reduced level of GH was seen during high levels of GIP.

Another potential mechanism is that alterations in ghrelin secretion from the stomach may contribute to the observed differences in GH dynamics during oral vs. IV glucose. Ghrelin release is stimulated by hypoglycaemia and suppressed by hyperglycaemia, and ghrelin stimulates GH secretion through direct action on somatotroph cells in the anterior pituitary [22]. No studies have directly compared circulating ghrelin levels during isoglycaemic oral vs. IV glucose administration. Thus, it is a possibility that oral glucose exposure may result in greater suppression of ghrelin and subsequent GH secretion compared to IV glucose. However, studies suggest that glucose suppresses ghrelin secretion without direct luminal contact to stomach mucosa, as most ghrelin-producing cells are ‘closed-type’ responding to basolateral signals, and suppression is also seen after parenteral glucose administration [10, 22–25].

An obvious limitation is that ghrelin was not measured, which prevents this study from making conclusions regarding a ghrelin mechanism in the suppression of GH. The study has a small sample size, which increases the risk of type II statistical errors and limits the generalizability of the findings. This limitation is particularly relevant for GH analyses, given the substantial inter-individual variability and pronounced intra-individual variation observed between the two test days as illustrated by the large error bars in Fig. 2. Ideally none of the subjects should have been treated with glucocorticoids. Though, we do not consider it a major confounder, as hydrocortisone dosing was consistent across both test days, and the plasma-glucose from the OGTT was mimicked very precisely during the intravenous glucose infusion. Finally, it cannot be excluded that the procedures related to parenteral administration of glucose could lead to a stress response resulting in transient increased levels of GH. However, the venous cannulas for administration of IV glucose were placed 15 min before the first GH measurement was done and during oral vs. IV glucose a venous cannula was used to obtain blood samples. Thus, we find it unlikely that changes in GH levels between the two interventions could fully be explained by changes in stress response.

An attempt to explore potential mechanisms could involve correlation analyses between GH and explanatory variables. However, we refrained from this approach because the dataset includes repeated measurements with short time intervals and pronounced dynamics, and importantly changes in one variable (e.g. glucose) may influence GH with a time lag. We therefore considered correlation analyses potentially misleading for investigating causality.

In conclusion, route of glucose exposure might influence the suppressive effect of glucose on GH secretion. The potential mechanism behind remains elusive but changes in other hormones might be of importance. More studies with e.g. inclusion of measurement of ghrelin and infusion of incretin hormones are needed to elucidate the topic and to confirm the results obtained in this small group of healthy subjects.

## Data Availability

Data is provided within the manuscript.
